# Knowledge, attitudes, and practices among patients with anemia towards disease management

**DOI:** 10.3389/fpubh.2024.1380710

**Published:** 2024-05-21

**Authors:** Binlian Yao, Min Xu, Fang Cheng, Min Peng, Xiaopei Mao

**Affiliations:** Department of Nursing, The First Affiliated Hospital of Zhejiang Chinese Medical University (Zhejiang Provincial Hospital of Chinese Medicine), Hangzhou, Zhejiang, China

**Keywords:** knowledge, attitudes, practices, anemia, cross-sectional study

## Abstract

**Objective:**

This study aimed to assess the knowledge, attitudes and practices among anemia patients toward disease management.

**Methods:**

This web-based cross-sectional study was conducted between September and December 2023 at The First Affiliated Hospital of Zhejiang Chinese Medical University (Zhejiang Provincial Hospital of Chinese Medicine). A self-designed questionnaire was developed to collect demographic information of anemia patients, and assess their knowledge, attitudes and practices (KAP) toward disease management.

**Results:**

A total of 396 valid questionnaires were collected. The mean age of the participants was 57.44 ± 16.80 years, and 52.02% were female. The mean knowledge, attitudes, and practices scores were 11.47 ± 1.73 (possible range: 0–14), 27.32 ± 2.96 (possible range: 7–35), and 40.49 ± 6.06 (possible range: 10–50), respectively. Multivariate analysis showed that bachelor’s degree or above was independently associated with sufficient knowledge (OR = 2.372, 95%CI: 1.160–4.853, *p* = 0.018). Knowledge (OR = 1.350, 95%CI: 1.166–1.563, *p* < 0.001) and hemoglobin within 60-90 g/L (OR = 1.782, 95%CI: 1.090–2.912, *p* = 0.021) were independently associated with positive attitudes. Moreover, attitudes (OR = 1.618, 95%CI: 1.454–1.799, *p* < 0.001) and diagnosis ≥1 year (OR = 1.949, 95%CI: 1.171–3.243, *p* = 0.010) were independently associated with proactive practices. The path analysis demonstrated that knowledge was directly and positively correlated with attitudes (β = 0.484, 95% CI: 0.363–0.647, *p* = 0.008), and attitudes was directly and positively correlated with practices (*β* = 1.195, 95% CI: 1.062–1.332, *p* = 0.007). Moreover, knowledge was indirectly and positively correlated with practice (*β* = 0.579, 95% CI: 0.434–0.805, *p* = 0.004).

**Conclusion:**

Anemia patients have sufficient knowledge, negative attitudes, but proactive practices toward the toward disease management Comprehensive training programs are needed to improve anemia patients practices in this area.

## Introduction

Anemia is characterized by a deficiency in the quantity or quality of red blood cells or hemoglobin in the blood (1). The reduced oxygen-carrying capacity of the blood impairs tissue oxygenation, which leads to symptoms such as dizziness, palpitations, and chest pain (2). Consequently, individuals with anemia may experience declines in physical performance (3), decreased productivity (4), and increased susceptibility to falls or accidents (5). Besides, population-based studies indicated that anemia can act as risk factors of severe complications like heart diseases, developmental delays in children and neurological development (6, 7).

Globally, anemia prevalence is a significant concern, particularly impacting developing countries. Approximately 1.62 billion people worldwide, corresponding to 24.8% of the global population, were affected by anemia in 2016 (8). This prevalence varied widely across different regions and populations, with higher rates observed in low- and middle-income countries, particularly in South Asia, Central and West Africa, and parts of Southeast Asia (9). Notably, the prevalence of anemia in sub-Saharan African countries elevated to the alarming 39% during the same period (10). In China, the prevalence varies widely across different regions and populations. For instance, among Chinese adults with newly diagnosed HIV/AIDS, the prevalence was 51.9% (11). Besides, in central and eastern China, it was 13.4% (12). In rural areas, the prevalence was reported at 9.7% (13). Of note, the prevalence of anemia was alarmingly high at 41.98% among pregnant women (14). These figures highlight the significant public health challenge posed by anemia in China.

Effective management of anemia involves more than just medical treatment, it requires active patient engagement in self-management. This includes understanding the condition (15), adhering to treatment plans (12, 16), and making lifestyle adjustments (17). A Knowledge, Attitudes, and Practices (KAP) study is a research methodology used to assess the understanding, beliefs, and behaviors of individuals regarding a specific health issue (18). In the context of anemia management, a KAP study aims to investigate patients’ knowledge about anemia, their attitudes towards the condition, and their practices in managing it. A prior KAP study indicated that Indian adolescent girls exhibited satisfactory knowledge and a moderate level of attitude towards anemia (19). However, their practice scores regarding anemia were found to be low to moderate (20). To date, no KAP evidence has been available on the Chinese patients with anemia, which greatly retards the improvement in treating and managing the condition.

In response to these existing research gaps, this study aimed to investigate the KAP among patients with anemia towards disease management. By elucidating the factors influencing the KAP towards anemia management, this research can contribute valuable insights into the development of patient-centered interventions. We hypothesized that the positive inter-relationships are found between each dimension of KAP. Our findings have implications for advancement of evidence-based interventions to enhance the quality of care and support provided to individuals living with anemia.

## Methods

### Study design and participants

This web-based cross-sectional study was conducted between September and December 2023 at The First Affiliated Hospital of Zhejiang Chinese Medical University (Zhejiang Provincial Hospital of Chinese Medicine). The study was ethically approved by the Ethics Committee of The First Affiliated Hospital of Zhejiang Chinese Medical University (Zhejiang Provincial Hospital of Chinese Medicine) (Approval No. 2023-KLS-273- 01) and informed consent was obtained from the study participants.

This study employed a convenience sampling method to recruit participants diagnosed with anemia. The inclusion criteria were as follows: (1) Diagnosis of anemia, (2) Age of 18 years or older, (3) Ability to independently read and complete the required questionnaires, or to do so with the researchers’ assistance, and (4) Willingness to participate in the study, evidenced by the provision of signed informed consent. Exclusion criteria encompassed individuals with impaired consciousness, mental disorders, or those exhibiting non-cooperation.

### Questionnaire

The questionnaire was developed with guidance from the relevant literature on anemia diagnosis and management (21–23). The initial draft was revised based on feedback from three experts in hematology and five experts in nursing of The First Affiliated Hospital of Zhejiang Chinese Medical University. Subsequently, a preliminary trial was conducted on a limited scale (*n* = 23), resulting in a Cronbach’s alpha coefficient value of 0.91, indicating good internal consistency.

The final questionnaire was in Chinese and consisted of four dimensions: demographic information, knowledge, attitudes and practices. The demographic information was consisted of 17 items, while the knowledge, attitude, and practice dimensions comprised 16, 7 and 10 items, respectively. Questions K11 and K13 were designed as trap questions, presenting exactly opposite meanings. They were used solely as a means to control the quality of the questionnaire and were not included in the subsequent statistical analysis. The study only provided a descriptive presentation of their correctness rates. Patients who selected “right” or “wrong” for both questions were deemed to have a logical conflict and were excluded from the survey. Consequently, the knowledge items were scored 1 point for a correct answer and 0 points for others, resulting in a possible score range of 0–14. The attitude items scored on a five-point Likert scale ranging from very positive (5 points) to very negative (1 point), with a possible score range of 7 to 35. The practice items also scored on a five-point Likert scale, ranging from very consistent (5 points) to very inconsistent (1 point), with a possible score range of 10 to 50.

Data collection was conducted via an online survey facilitated by the Sojump platform.^1^ The survey was disseminated to participants by QR codes linking to the questionnaire in various hospital settings, including wards, consultation rooms, and offices of medical staff. Prior to accessing the survey questions, participants were required to select the option “I agree to participate in this study.” This process ensured informed consent. All responses were gathered anonymously. To prevent duplicate submissions, an IP address restriction was implemented, allowing only one completed survey per unique IP address.

### Statistical methods

STATA 17.0 (Stata Corporation, College Station, TX, United States) was used for statistical analysis. The continuous variables were expressed as mean ± SD, and the categorical variables was expressed as *N* (%). The continuous variables conformed to a normal distribution were tested by the *t*-test or ANOVA. Bloom’s cut-off of 80% was used to determine sufficient knowledge (≥11.2 points), positive attitude (≥28 points), and proactive practice (≥40) as the binary outcomes (24). Variables with *p* < 0.05 in the univariate logistic regression analysis are included in the multivariate logistic regression analysis (19). Pearson correlation analysis was used to analyze the correlation between KAP. The path analysis of KAP among anemia patients toward disease management was constructed with AMOS 24.0 (IBM, NY, United States). The hypotheses as following: (1) knowledge had direct effects on attitude, (2) knowledge had direct effects on practice, and (3) attitude had direct effects on practice.

## Results

The initial survey generated a total of 500 responses. Among these, 104 of these questionnaires were subsequently excluded due to logical inconsistencies in the responses. As a result, the study successfully gathered 396 valid questionnaires, constituting an effective response rate of 79.20%.

The mean age of the participants was 57.44 ± 16.80 years. The majority were female (52.02%), and had BMI (kg/m^2^) within the range of 18.5–23.9 kg m^−2^ (56.06%). Furthermore, a significant proportion resided in rural areas (54.04%), possessed an educational background of junior high school or below (65.15%), and were covered by Urban Resident Basic Medical Insurance (URBMI). Additionally, 38.64% were retired, and 42.93% reported a monthly income ranging from 2,000 to 5,000 CNY. Health-related behaviors included the majority abstaining from both smoking (89.65%) and alcohol consumption (89.39%). Regarding hemoglobin levels, 39.14% exhibited levels within the 60-90 g/L range, while 38.13% recorded hemoglobin levels exceeding 90 g/L but below the normal threshold. Besides, a majority (55.81%) had undergone blood transfusions. Furthermore, 44.70% received an anemia diagnosis within the past year, while a slightly lower percentage (43.94%) received a diagnosis more than a year before (Table 1).

**Table 1:** Demographic characteristics.

	*N* (%)	Knowledge score		Attitude score		Practice score	
		Mean ± SD	*p*	Mean ± SD	*p*	Mean ± SD	*P*
Total	396	11.47 ± 1.73		27.32 ± 2.96		40.49 ± 6.06	
Gender			0.316		0.263		0.597
Male	190 (47.98)	11.56 ± 1.58		27.49 ± 2.94		40.66 ± 5.99	
Female	206 (52.02)	11.39 ± 1.86		27.16 ± 2.98		40.34 ± 6.14	
Age (years)	57.44 ± 16.80						
BMI (kg/m^2^)			0.738		0.144		0.055
<18.5	57 (14.39)	11.51 ± 1.95		26.91 ± 3.21		39.07 ± 6.50	
18.5–23.9	222 (56.06)	11.52 ± 1.70		27.19 ± 2.95		40.39 ± 6.08	
≥24.0	117 (29.55)	11.37 ± 1.68		27.74 ± 2.84		41.39 ± 5.70	
Residence			0.220		0.036		0.016
Rural	214 (54.04)	11.37 ± 1.86		27.03 ± 3.00		39.82 ± 6.21	
Urban	182 (45.96)	11.59 ± 1.56		27.65 ± 2.90		41.29 ± 5.81	
Marital status			0.389		0.826		0.428
Single	73 (18.43)	11.63 ± 1.59		27.25 ± 3.18		39.99 ± 7.07	
Married	323 (81.57)	11.44 ± 1.76		27.33 ± 2.92		40.61 ± 5.82	
Highest education			<0.001		<0.001		0.086
Junior high school or below	258 (65.15)	11.22 ± 1.86		26.91 ± 3.06		40.01 ± 5.74	
High school or technical school	46 (11.62)	11.43 ± 1.83		27.41 ± 2.67		41.07 ± 6.29	
College and above	92 (23.23)	12.18 ± 0.95		28.40 ± 2.56		41.57 ± 6.72	
Occupation			0.005		0.015		0.493
Formal employee/ part-time/ self-employed	85 (21.46)	12.06 ± 1.22		28.20 ± 2.76		41.38 ± 6.78	
Unemployed	38 (9.60)	11.39 ± 1.92		26.71 ± 2.70		40.03 ± 5.66	
Retired	153 (38.64)	11.27 ± 1.82		27.09 ± 3.04		40.37 ± 5.71	
Other	120 (30.30)	11.33 ± 1.79		27.17 ± 3.00		40.18 ± 6.10	
Monthly *per capita* income (CNY)			0.018		0.034		0.399
<2,000	125 (31.57)	11.40 ± 1.85		27.00 ± 2.85		40.07 ± 6.15	
2,000–5,000	170 (42.93)	11.25 ± 1.77		27.14 ± 3.15		40.28 ± 5.86	
5,001-10,000	66 (16.67)	11.92 ± 1.35		27.79 ± 2.56		41.42 ± 6.34	
>10,000	35 (8.84)	11.94 ± 1.53		28.43 ± 2.87		41.29 ± 6.19	
Health insurance			0.466		0.448		0.797
No health insurance	3 (0.76)	11.67 ± 1.53		26.00 ± 2.65		39.00 ± 9.17	
New Rural Cooperative Medical Scheme (NRCMS)	139 (35.10)	11.27 ± 1.87		27.11 ± 3.14		40.19 ± 6.07	
Urban Resident Basic Medical Insurance (URBMI)	240 (60.61)	11.59 ± 1.65		27.42 ± 2.88		40.61 ± 6.05	
Commercial insurance	5 (1.26)	11.80 ± 1.10		26.60 ± 1.67		43.20 ± 1.48	
Other	9 (2.27)	11.11 ± 1.90		28.67 ± 2.92		41.00 ± 7.45	
Comorbidities (Multiple choices)							
Malnutrition	40 (10.10)	11.70 ± 1.60		26.83 ± 3.10		39.25 ± 6.19	
Chronic kidney disease	52 (13.13)	11.40 ± 1.62		27.62 ± 3.09		41.23 ± 5.87	
Bleeding	15 (3.79)	10.93 ± 2.25		27.40 ± 3.00		39.80 ± 5.53	
Aplastic anemia	95 (23.99)	11.52 ± 1.79		27.87 ± 2.82		42.67 ± 5.78	
Hereditary anemia (e.g., thalassemia, sickle cell anemia)	5 (1.26)	11.00 ± 1.73		28.20 ± 3.27		40.40 ± 7.44	
Autoimmune diseases	11 (2.78)	11.73 ± 0.79		26.64 ± 2.29		37.55 ± 9.52	
Cancer and radiotherapy	123 (31.06)	11.33 ± 1.82		26.68 ± 3.04		39.65 ± 6.13	
Anemia during pregnancy	3 (0.76)	12.33 ± 1.15		30.33 ± 1.15		49.00 ± 1.73	
Myelodysplastic syndromes	41 (10.35)	11.44 ± 1.70		27.90 ± 2.81		41.59 ± 5.64	
Other	73 (18.43)	11.62 ± 1.60		26.79 ± 2.92		39.38 ± 5.17	
None of the above	12 (3.03)	12.58 ± 0.79		27.00 ± 2.89		38.75 ± 6.12	
Smoking			0.484		0.741		0.537
Yes	41 (10.35)	11.29 ± 1.81		27.17 ± 2.99		41.05 ± 5.04	
No	355 (89.65)	11.49 ± 1.72		27.33 ± 2.96		40.43 ± 6.17	
Alcohol consumption			0.863		0.901		0.606
Yes	42 (10.61)	11.43 ± 1.89		27.26 ± 3.50		40.95 ± 5.04	
No	354 (89.39)	11.48 ± 1.71		27.32 ± 2.90		40.44 ± 6.18	
Habit of regular exercise			0.891		0.897		0.103
Yes	135 (34.09)	11.49 ± 1.67		27.29 ± 2.91		41.19 ± 5.60	
No	261 (65.91)	11.46 ± 1.77		27.33 ± 3.00		40.14 ± 6.27	
Range of most recent hemoglobin test			0.460		0.079		0.161
>90 g/L, but below normal	151 (38.13)	11.62 ± 1.70		26.93 ± 3.03		39.85 ± 6.31	
60-90 g/L	155 (39.14)	11.37 ± 1.85		27.61 ± 2.86		40.63 ± 6.06	
<60 g/L	63 (15.91)	11.29 ± 1.59		27.13 ± 2.96		40.79 ± 5.06	
Normal (female>110 g/L male>120 g/L)	27 (6.82)	11.63 ± 1.55		28.22 ± 2.97		42.59 ± 6.57	
History of blood transfusion			0.878		0.428		0.023
Yes	221 (55.81)	11.48 ± 1.69		27.42 ± 2.90		41.11 ± 5.85	
No	175 (44.19)	11.46 ± 1.78		27.18 ± 3.05		39.72 ± 6.25	
Date of anemia diagnosis			0.499		0.185		0.004
Less than 1 year	177 (44.70)	11.56 ± 1.73		27.53 ± 2.91		40.34 ± 6.15	
1 year and more	174 (43.94)	11.36 ± 1.72		27.28 ± 2.97		41.30 ± 5.88	
Uncertain	45 (11.36)	11.58 ± 1.76		26.62 ± 3.11		37.98 ± 5.77	

In the items of health insurance, No Health Insurance refers to individuals who do not have any form of health insurance coverage. New Rural Cooperative Medical Scheme (NRCMS) is aimed at rural residents who are not formally employed. It is a voluntary program funded by individual premiums (subsidized by the government) and government contributions. The NRCMS primarily covers serious illnesses and provides a basic medical safety net, but coverage is limited, and out-of-pocket costs can still be significant. Urban Resident Basic Medical Insurance (URBMI) targets non-working urban residents, including children, the elderly, and others without formal employment. Similar to NRCMS, it is funded by personal contributions and government subsidies. The URBMI covers both inpatient and outpatient services, though the extent of coverage can vary by locality. Commercial Insurance is private health insurance purchased from commercial insurance companies. Individuals often buy commercial insurance to supplement the basic coverage provided by NRCMS or URBMI, as these programs can have substantial coverage gaps. Other insurance may include various other forms of insurance coverage which are not as commonly utilized or are specific to certain groups of people, such as employee health benefits provided by some companies, insurance schemes for government employees, or local health insurance programs that may exist in specific areas.

The mean knowledge, attitudes, and practices scores were 11.47 ± 1.73 (possible range: 0–14), 27.32 ± 2.96 (possible range: 7–35), and 40.49 ± 6.06 (possible range: 10–50), respectively. High knowledge scores were found among participants with a higher educational level (*p* < 0.001), those engaged in formal employment, part-time work, or self-employment (*p* = 0.005), and those with a monthly income exceeding 10,000 CNY (*p* = 0.018). Higher attitude scores were evident among participants residing in urban areas (*p* = 0.036), those with a higher educational level (*p* < 0.001), individuals engaged in formal employment, part-time work, or self-employment (*p* = 0.015), and those with a monthly income exceeding 10,000 CNY (*p* = 0.034). Besides, higher practice scores were observed among participants residing in urban areas (*p* = 0.016), those who had undergone a blood transfusion (*p* = 0.023), and individuals with anemia for 1 year or more (*p* = 0.004) (Table 1).

The highest correctness rates among the three knowledge items were observed for the statements: “Individuals with anemia typically exhibit pale skin” (K6) at 94.44%, “Treatment options for anemia may encompass iron supplements, blood transfusions, and hematopoietic growth factors” (K15) at 92.93%, and “Symptoms of anemia may include headaches, tinnitus, and cerebral hypoxia” (K7) at 92.17%. Conversely, the three items with the lowest correctness rates were: “Chemical substances such as benzene, herbicides, and pesticides can induce anemia” (K3) with a correctness rate of 38.13%, “While causes of anemia may vary, the treatment principles remain consistent” (K12) at 44.19%, and “Insufficient intake of iron, folic acid, and vitamin B12 leading to anemia can result from an imbalanced diet, poor cooking habits, and gastrointestinal diseases” (K2) at 82.07% (Supplementary Table S1).

A significant majority (96.47%) expressed a positive attitude, emphasizing the importance of adhering to medical advice, attending regular follow-up appointments, and following prescribed medication regimens (A4). In contrast, the lowest proportion (72.72%) indicated anxiety regarding the physical and mental discomfort associated with anemia and the potential for serious complications (A6). Likewise, 76.52% expressed full confidence in their ability to strictly adhere to self-management practices for anemia (A7) (Supplementary Table S2).

The highest proportion (92.93%) actively participated in the treatment of the underlying disease for improved disease management (P9). In contrast, only 58.84% recognized that the disease induces negative emotions like depression and anxiety, prompting them to actively seek psychological counseling or other forms of support (P8). Moreover, 60.10% actively sought information about anemia in the past year (P1) (Supplementary Table S3).

Pearson’s analysis was performed to assess the relationship between knowledge, attitudes, and practices. It was shown that the knowledge and the attitudes were positively correlated (*r* = 0.283, *p* < 0.001), and knowledge and practices were also positively correlated (*r* = 0.236, *p* < 0.001). Additionally, there was a positive correlation between attitude and practice scores (*r* = 0.604, *p* < 0.001) (Supplementary Table S4).

Multivariate analysis showed that bachelor’s degree or above was positively associated with knowledge (OR = 2.372, 95%CI: 1.160–4.853, *p* = 0.018) (Table 2). Besides, knowledge (OR = 1.350, 95%CI: 1.166–1.563, *p* < 0.001) and hemoglobin within 60-90 g/L (OR = 1.782, 95%CI: 1.090–2.912, *p* = 0.021) were positively associated with attitude. However, unemployment was negatively associated with attitude (OR = 0.416, 95%CI: 0.173–0.996, *p* = 0.049) (Table 3). Moreover, attitude (OR = 1.618, 95%CI: 1.454–1.799, *p* < 0.001) and diagnosis ≥1 year (OR = 1.949, 95%CI: 1.171–3.243, *p* = 0.010) were positively associated with practice (Table 4).

**Table 2:** Multivariable analysis of the knowledge.

	Univariable analysis		Multivariable analysis	
	OR(95%CI)	*p*	OR(95%CI)	*p*
Gender				
Male	ref.			
Female	0.812(0.541 1.218)	0.313		
Age (years)	0.981(0.969 0.993)	0.003	0.992(0.973 1.010)	0.381
BMI (kg/m^2^)				
<18.5	ref.			
18.5–23.9	0.733(0.397 1.353)	0.321		
≥24.0	0.800(0.411 1.555)	0.511		
Residence				
Rural	ref.			
Urban	1.282(0.853 1.926)	0.232		
Marital status				
Single	ref.			
Married	0.727(0.425 1.244)	0.245		
Highest education				
Junior high school or below	ref.		ref.	
High school or technical school	1.797(0.926 3.488)	0.083	1.452(0.708 2.979)	0.309
College and above	3.341(1.907 5.584)	<0.001	2.372(1.160 4.853)	0.018
Occupation				
Formal employee/ part-time/ self-employed	ref.		ref.	
Unemployed	0.451(0.201 1.015)	0.054	0.535(0.220 1.303)	0.168
Retired	0.469(0.260 0.845)	0.012	0.915(0.439 1.908)	0.813
Other	0.401(0.218 0.738)	0.003	0.650(0.316 1.337)	0.242
Monthly *per capita* income (CNY)				
<2,000	ref.		ref.	
2,000–5,000	0.963(0.605 1.535)	0.875	0.756(0.443 1.291)	0.306
5,001-10,000	2.192(1.138 4.222)	0.019	1.178(0.551 2.515)	0.673
>10,000	2.567(1.081 6.095)	0.033	1.087(0.399 2.966)	0.870
Smoking				
Yes	0.794(0.413 1.524)	0.487		
No	ref.			
Alcohol consumption				
Yes	0.928(0.484 1.782)	0.823		
No	ref.			
Habit of regular exercise				
Yes	1.074(0.700 1.646)	0.744		
No	ref.			
Range of most recent hemoglobin test				
>90 g/L, but below normal	ref.			
60-90 g/L	0.688(0.433 1.094)	0.114		
<60 g/L	0.726(0.397 1.328)	0.299		
Normal (female>110 g/L male>120 g/L)	0.742(0.321 1.716)	0.485		
History of blood transfusion				
Yes	1.042(0.694 1.564)	0.845		
No	ref.			
Date of anemia diagnosis				
Less than 1 year	ref.			
1 year and more	0.712(0.463 1.095)	0.122		
Uncertain	0.888(0.451 0.748)	0.731		

The regression model for multivariate logistic regression analysis was: Knowledge ~ Age + Highest education + Occupation + Monthly per capita income.

**Table 3:** Multivariable analysis of the attitudes.

	Univariable analysis		Multivariable analysis	
	OR(95%CI)	*p*	OR(95%CI)	*p*
Knowledge score	1.364(1.186 1.568)	<0.001	1.350(1.166 1.563)	<0.001
Gender				
Male	ref.			
Female	0.841(0.560 1.263)	0.403		
Age (years)	0.989(0.977 1.001)	0.067		
BMI (kg/m^2^)				
<18.5	ref.			
18.5–23.9	1.042(0.567 1.916)	0.894		
≥24.0	1.381(0.717 2.660)	0.335		
Residence				
Rural	ref.			
Urban	1.192(0.793 1.790)	0.399		
Marital status				
Single	ref.			
Married	1.212(0.712 2.065)	0.479		
Highest education				
Junior high school or below	ref.		ref.	
High school or technical school	0.903(0.463 1.761)	0.765	0.796(0.384 1.649)	0.539
College and above	1.787(1.103 2.895)	0.018	1.312(0.749 2.298)	0.343
Occupation				
Formal employee/ part-time/ self-employed	ref.		ref.	
Unemployed	0.366(0.158 0.845)	0.019	0.416(0.173 0.996)	0.049
Retired	0.543(0.316 0.931)	0.027	0.725(0.390 1.348)	0.309
Other	0.614(0.350 1.079)	0.090	0.779(0.409 1.482)	0.447
Monthly *per capita* income (CNY)				
<2,000	ref.			
2,000–5,000	1.193(0.736 1.935)	0.474		
5,001-10,000	1.368(0.739 2.531)	0.318		
>10,000	1.866(0.873 3.990)	0.107		
Smoking				
Yes	1.056(0.544 2.049)	0.873		
No	ref.			
Alcohol consumption				
Yes	1.406(0.738 2.680)	0.300		
No	ref.			
Habit of regular exercise				
Yes	0.860(0.559 1.324)	0.493		
No	ref.			
Range of most recent hemoglobin test				
>90 g/L, but below normal	ref.		ref.	
60-90 g/L	1.550(0.970 2.475)	0.067	1.782(1.090 2.912)	0.021
<60 g/L	1.234(0.666 2.286)	0.504	1.407(0.740 2.676)	0.297
Normal (female>110 g/L male>120 g/L)	2.311(1.009 5.294)	0.048	2.237(0.928 5.391)	0.073
History of blood transfusion				
Yes	1.058(0.702 1.593)	0.788		
No	ref.			
Date of anemia diagnosis				
Less than 1 year	ref.			
1 year and more	0.807(0.524 1.243)	0.331		
Uncertain	0.805(0.408 1.588)	0.531		

The regression model for multivariate logistic regression analysis was: Attitude ~ Knowledge + Highest education + Occupation + Range of most recent hemoglobin test.

**Table 4:** Multivariable analysis of the practices.

	Univariable analysis		Multivariable analysis	
	OR(95%CI)	*p*	OR(95%CI)	*p*
Knowledge score	1.267(1.121 1.433)	<0.001	1.123(0.969 1.301)	0.124
Attitude score	1.612(1.457 1.784)	<0.001	1.618(1.454 1.799)	<0.001
Gender				
Male	ref.			
Female	0.922(0.622 1.368)	0.687		
Age (years)	0.989(0.978 1.001)	0.076		
BMI (kg/m^2^)				
<18.5	ref.			
18.5–23.9	1.303(0.724 2.345)	0.378		
≥24.0	1.779(0.938 3.376)	0.078		
Residence				
Rural	ref.			
Urban	1.226(0.825 1.821)	0.314		
Marital status				
Single	ref.			
Married	1.106(0.665 1.839)	0.698		
Highest education				
Junior high school or below	ref.			
High school or technical school	1.143(0.610 2.141)	0.677		
College and above	1.143(0.710 1.840)	0.583		
Occupation				
Formal employee/ part-time/ self-employed	ref.			
Unemployed	0.932(0.434 2.003)	0.856		
Retired	0.873(0.513 1.484)	0.615		
Other	0.963(0.553 1.680)	0.895		
Monthly *per capita* income (CNY)				
<2,000	ref.			
2,000–5,000	1.040(0.656 1.651)	0.867		
5,001-10,000	0.956(0.527 1.737)	0.883		
>10,000	1.076(0.508 2.277)	0.848		
Smoking				
Yes	0.947(0.496 1.808)	0.869		
No	ref.			
Alcohol consumption				
Yes	1.000(0.527 1.896)	1.000		
No	ref.			
Habit of regular exercise				
Yes	1.340(0.883 2.034)	0.169		
No	ref.			
Range of most recent hemoglobin test				
>90 g/L, but below normal	ref.			
60-90 g/L	1.336(0.852 2.094)	0.207		
<60 g/L	1.343(0.745 2.420)	0.327		
Normal (female>110 g/L male>120 g/L)	1.775(0.773 4.079)	0.176		
History of blood transfusion				
Yes	1.418(0.952 2.111)	0.086		
No	ref.			
Date of anemia diagnosis				
Less than 1 year	ref.		ref.	
1 year and more	1.463(0.960 2.228)	0.077	1.949(1.171 3.243)	0.010
Uncertain	0.440(0.216 0.893)	0.023	0.451(0.195 1.045)	0.063

The regression model for multivariate logistic regression analysis was: Practice ~ Knowledge + Attitude + Date of anemia diagnosis.

**Figure 1:**
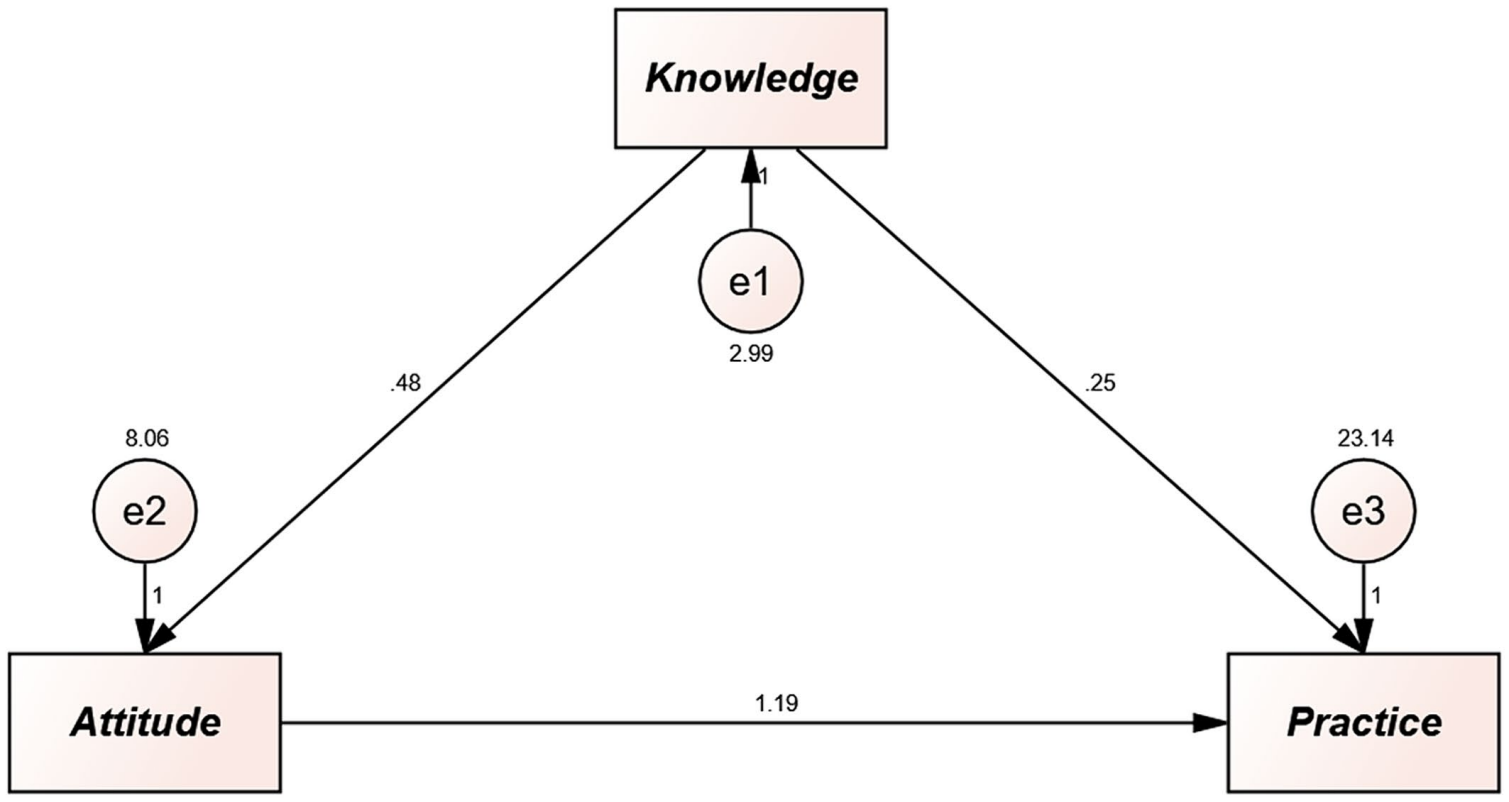
Path analysis showing the associations between KAP scores. All variables are observed variables. Direction of causality is indicated by single-headed arrows. The standardized path coefficients are presented alongside the arrows.

Path analysis was conducted to explore the direct and indirect relationships among KAP scores. The path analysis demonstrated that knowledge was directly and positively correlated with attitudes (*β* = 0.484, 95% CI: 0.363–0.647, *p* = 0.008), and attitudes was directly and positively correlated with practices (*β* = 1.195, 95% CI: 1.062–1.332, *p* = 0.007). Moreover, knowledge was indirectly and positively correlated with practice (*β* = 0.579, 95% CI: 0.434–0.805, *p* = 0.004) (Supplementary Table S5; Figure 1).

## Discussion

The findings revealed that anemia patients have sufficient knowledge, negative attitudes, and proactive practices toward the disease management. Positive associations were observed among KAP scores. Comprehensive training programs are needed to improve anemia patients practices in this area.

Reportedly, Indian adolescent girls had similarly good knowledge and medium level of attitude towards anemia (20). Besides, low to moderate KAP scores towards anemia were observed among Indonesian adolescent girls (25). The shared negative attitudes in both published and our findings may stem from the chronicity of the condition, stigma or misconceptions associated with the anemia, and the perceived burden of long-term management. Therefore, patient-centered care models are recommended to address not only the physiological aspects of the condition, but also the psychosocial dimensions through open communication and shared decision-making. Of note, the high levels of knowledge and practice can lay strong foundations for psychosocial interventions.

As high as 94.44% correctly identified the pale skin as the symptom of anemia, which aligned with established medical knowledge (26). Moreover, the awareness of pale skin extends beyond mere knowledge, since the condition plays a pivotal role in early detection and timely intervention (27). However, only 38.13% exhibited the awareness regarding the potential environmental factors contributing to anemia. Environmental exposures to substances like benzene, herbicides, and pesticides have been documented to pose health risks, and their potential link to anemia has been explored in scientific literature (28–30). The underlying mechanisms include damaging the DNA in blood-forming cells, interfering with hormonal systems, disrupting endocrine function, or causing cellular damage (28–30). This finding has significant implications for public health education to address awareness gaps related to the etiological factors of anemia. Besides, a notable misconception was found among the participants regarding the uniformity of treatment principles for anemia (44.19%). In the anemia management, understanding the heterogeneity of causative factors and tailoring treatments accordingly is fundamental (31). The lack of awareness regarding the variation in treatment principles might stem from oversimplified health messaging or inadequate dissemination of comprehensive information to the public.

A majority of anemia patients (96.47%) had positive attitude toward adhering to medical advice, attending regular follow-up appointments, and following prescribed medication regimens. The positive attitude can be interpreted as a manifestation of patient engagement and a willingness to actively participate in their own healthcare. Such attitudes have been associated with improved treatment adherence, better management of chronic conditions, and overall positive health outcomes (32). Besides, 72.72% expressed anxiety about the physical and mental discomfort associated with anemia and the potential for serious complications. The anxiety reported by anemia patients may be attributed to various factors, including concerns about the impact of anemia on daily life, fear of complications, and uncertainty about the course of the condition. The finding underscored the importance of integrating both hematologists and mental health professionals into the overall care plan, as proposed by collaborative care models (33). Moreover, the high level of self-confidence reported by 76.52% in their ability to strictly adhere to self-management practices for anemia reflected their engagement in the healthcare. Encouraging and supporting this self-efficacy can contribute to better adherence to treatment plans, improved health outcomes, and enhanced overall well-being. Future research should continue to explore the dynamics of patient confidence and its impact on long-term anemia management.

As high as 92.93% actively participated in the treatment of the underlying disease for improved disease management. The current study’s higher engagement rate suggests advancements in healthcare provider-patient communication (34). Whereas, only 58.84% acknowledged that the psychosocial implications of anemia prompted the subsequent seeking of support. It highlighted that acknowledgment of psychosocial implications did not necessarily translate into active support-seeking behavior. This raised questions about potential barriers preventing individuals from seeking the necessary support. These barriers could include stigmas associated with mental health, lack of accessible support systems, or insufficient integration of psychosocial care into routine anemia management (35). Moreover, the observed rate of 60.10% actively seeking information in the past year reflected the prevalent engagement in understanding and addressing the anemia. Web-based health information seeking has been established to play important roles in the management of different disease (36). It can be recommended to promote online educational initiatives, empowering patients with the knowledge needed to make informed decisions, adhere to treatment plans, and actively participate in disease management.

Correlation analysis demonstrated the positive correlations among KAP scores, which aligned with the health belief model (37). In other words, anemia patients with greater knowledge are more likely to perceive the severity of their condition and engage in behaviors to address it. The influential factors of KAP scores were identified in the multivariate analysis. First, individuals with bachelor’s degree or above were more likely to possess knowledge about anemia. This aligned with the published literature indicating that education is a key determinant of health knowledge, as individuals with higher educational backgrounds tend to have better access to information and resources (38). Second, lower hemoglobin levels within the range of 60–90 g/L were positively associated with attitudes. Symptoms such as fatigue, weakness, and reduced quality of life may contribute to elevated attitudes, such as increased concerns about the condition, emotional distress, or altered perceptions of disease impact (39). Third, the negative association between unemployment and attitudes was observed. Unemployment can lead to financial strain, increased stress levels, and reduced access to healthcare resources, all of which may contribute to negative attitudes towards the management of anemia (40).

### Limitations of the study

The study had several limitations. Initially, the cross-sectional design imposed challenges in establishing causal relationships in our findings. Secondly, the reliance on self-reported data introduces the risk of social desirability bias, which may lead to artificially elevated scores (41).

### Areas for further research

Anemia patients have sufficient knowledge, negative attitudes, and proactive practices toward the disease management. Future research with larger sample size and multi-center design is needed to verify our findings. Besides, due to the cross-sectional design, longitudinal studies are warranted to infer causal relationships of KAP and possible interventional effects.

## Conclusion

Despite its limitations, this study provides essential insights into the disease management dynamics of anemia patients. Utilizing path and Pearson correlation analyses, as well as univariate and multivariate statistical methods, it affirmed the relevance of the KAP of disease management among patients with anemia. Moreover, the examination of demographic and clinical factors further informs the development of these interventions. Such strategies are poised to amplify patient knowledge, which is pivotal in promoting positive attitudes and proactive behaviors, ultimately enhancing health outcomes.

## Data availability statement

The original contributions presented in the study are included in the article/Supplementary material, further inquiries can be directed to the corresponding author.

## Ethics statement

The studies involving humans were approved by The First Affiliated Hospital of Zhejiang Chinese Medical University (Zhejiang Provincial Hospital of Chinese Medicine) [No. 2023-KLS-273- 01]. The studies were conducted in accordance with the local legislation and institutional requirements. The participants provided their written informed consent to participate in this study.

## Author contributions

BY: Writing – review & editing, Writing – original draft, Data curation. MX: Writing – review & editing, Writing – original draft, Formal analysis. FC: Writing – review & editing, Writing – original draft, Data curation. MP: Writing – review & editing, Writing – original draft, Data curation. XM: Writing – review & editing, Writing – original draft, Data curation.

## Footnotes

^1^ http://www.sojump.com

## References

[1] Garcia-Casal MN Dary O Jefferds ME Pasricha SR. Diagnosing anemia: challenges selecting methods, addressing underlying causes, and implementing actions at the public health level. Ann N Y Acad Sci. (2023) 1524:37–50. doi: 10.1111/nyas.14996, PMID: 37061792 PMC10880862

[2] Hanna-Rivero N Tu SJ Elliott AD Pitman BM Gallagher C Lau DH. Anemia and iron deficiency in patients with atrial fibrillation. BMC Cardiovasc Disord. (2022) 22:204. doi: 10.1186/s12872-022-02633-6, PMID: 35508964 PMC9066804

[3] Marzban M Nabipour I Farhadi A Ostovar A Larijani B Darabi AH. Association between anemia, physical performance and cognitive function in Iranian elderly people: evidence from Bushehr elderly health (BEH) program. BMC Geriatr. (2021) 21:329. doi: 10.1186/s12877-021-02285-9, PMID: 34030664 PMC8142505

[4] Marcus H Schauer C Zlotkin S. Effect of Anemia on work productivity in both labor-and nonlabor-intensive occupations: a systematic narrative synthesis. Food Nutr Bull. (2021) 42:289–308. doi: 10.1177/03795721211006658, PMID: 33874760

[5] Thaler-Kall K Döring A Peters A Thorand B Grill E Koenig W. Association between anemia and falls in community-dwelling older people: cross-sectional results from the KORA-age study. BMC Geriatr. (2014) 14:29. doi: 10.1186/1471-2318-14-29, PMID: 24602338 PMC3973957

[6] Xia H Shen H Cha W Lu Q. The prognostic significance of anemia in patients with heart failure: a meta-analysis of studies from the last decade. Front Cardiovasc Med. (2021) 8:632318. doi: 10.3389/fcvm.2021.632318, PMID: 34055927 PMC8155282

[7] Zheng J Liu J Yang W. Association of iron-deficiency anemia and non-iron-deficiency anemia with neurobehavioral development in children aged 6–24 months. Nutrients. (2021) 13:3423. doi: 10.3390/nu13103423, PMID: 34684422 PMC8537382

[8] Shah SA Soomro U Ali O Tariq Y Waleed MS Guntipalli P. The prevalence of Anemia in working women. Cureus. (2023) 15:e44104. doi: 10.7759/cureus.44104, PMID: 37750111 PMC10518160

[9] Sunuwar DR Singh DR Chaudhary NK Pradhan PMS Rai P Tiwari K. Prevalence and factors associated with anemia among women of reproductive age in seven south and southeast Asian countries: evidence from nationally representative surveys. PLoS One. (2020) 15:e0236449. doi: 10.1371/journal.pone.0236449, PMID: 32790764 PMC7425935

[10] Zegeye B Anyiam FE Ahinkorah BO Ameyaw EK Budu E Seidu A-A. Prevalence of anemia and its associated factors among married women in 19 sub-Saharan African countries. Arch Public Health. (2021) 79:214. doi: 10.1186/s13690-021-00733-x, PMID: 34839822 PMC8628470

[11] Shen Y Wang Z Lu H Wang J Chen J Liu L. Prevalence of anemia among adults with newly diagnosed HIV/AIDS in China. PLoS One. (2013) 8:e73807. doi: 10.1371/journal.pone.0073807, PMID: 24058490 PMC3776781

[12] Li L Luo R Medina A Rozelle S. The prevalence of ANEMIA in central and eastern China: evidence from the China health and nutrition survey. Southeast Asian J Trop Med Public Health. (2015) 46:306–21. PMID: 26513934

[13] Li M Hu Y Mao D Wang R Chen J Li W. Prevalence of Anemia among Chinese rural residents. Nutrients. (2017) 9:192. doi: 10.3390/nu9030192, PMID: 28245576 PMC5372855

[14] Hu H Huang A Yang Q Zhao W Ma Y Di J. Prevalence and risk factors of Anemia of pregnant women - 6 provinces in China, 2014-2018. China CDC Wkly. (2020) 2:225–9. doi: 10.46234/ccdcw2020.058 34594628 PMC8428443

[15] Tim Goodnough L Comin-Colet J Leal-Noval S Ozawa S Takere J Henry D. Management of anemia in patients with congestive heart failure. Am J Hematol. (2017) 92:88–93. doi: 10.1002/ajh.24595 27779769

[16] Shander A Javidroozi M Lobel G. Patient blood Management in the Intensive Care Unit. Transfus Med Rev. (2017) 31:264–71. doi: 10.1016/j.tmrv.2017.07.007 28811051

[17] Mildon A Lopez de Romaña D Jefferds MED Rogers LM Golan JM Arabi M. Integrating and coordinating programs for the management of anemia across the life course. Ann N Y Acad Sci. (2023) 1525:160–72. doi: 10.1111/nyas.15002, PMID: 37194608 PMC10918752

[18] Kwol VS Eluwole KK Avci T Lasisi TT. Another look into the knowledge attitude practice (KAP) model for food control: an investigation of the mediating role of food handlers’ attitudes. Food Control. (2020) 110:107025. doi: 10.1016/j.foodcont.2019.107025

[19] Li C Meng Y Meng X Song Y. Knowledge, attitude and practice toward oral anticoagulants among patients with atrial fibrillation. Front Cardiovasc Med. (2023) 10:1301442. doi: 10.3389/fcvm.2023.1301442, PMID: 38162125 PMC10755671

[20] Dhivakar M Iswariya M Jobin PC. The knowledge and attitude towards anaemia amongst adolescent girls. Indian J Contin Nurs Educ. (2020) 21:100–3. doi: 10.4103/IJCN.IJCN_30_20

[21] Freeman AM Rai M Morando DW. Anemia screening. In: StatPearls [Internet]. Treasure Island, Florida: StatPearls Publishing (2018). 29763080

[22] Provenzano R Lerma EV Szczech L. Management of anemia: a comprehensive guide for clinicians. Memphis Med J. (2018) 22. Available at: https://link.springer.com/book/10.1007/978-1-4939-7360-6

[23] Robalo Nunes A Mairos J Brilhante D Marques F Belo A Cortez J. Screening for anemia and iron deficiency in the adult Portuguese population. Anemia. (2020) 2020:1–10. doi: 10.1155/2020/1048283, PMID: 32802501 PMC7411453

[24] Olum R Chekwech G Wekha G Nassozi DR Bongomin F. Coronavirus disease-2019: knowledge, attitude, and practices of health care workers at Makerere University teaching hospitals, Uganda. Front Public Health. (2020) 8:181. doi: 10.3389/fpubh.2020.00181, PMID: 32426320 PMC7204940

[25] Agustina R Wirawan F Sadariskar AA Setianingsing AA Nadiya K Prafiantini E. Associations of knowledge, attitude, and practices toward Anemia with Anemia prevalence and height-for-age Z-score among Indonesian adolescent girls. Food Nutr Bull. (2021) 42:S92–s108. doi: 10.1177/03795721211011136, PMID: 34282657

[26] Bhadra PAD. A review on nutritional anemia. Indian J Nat Sci. (2020) 10:18466–74.

[27] Kakkar M Holderle K Sheth M Arany S Schiff L Planerova A. Orofacial manifestation and dental Management of Sickle Cell Disease: a scoping review. Anemia. (2021) 2021:5556708–8. doi: 10.1155/2021/5556708, PMID: 34721900 PMC8556080

[28] Bunsri S Muenchamnan N Naksen W Ong-Artborirak P. The hematological and biochemical effects from pesticide exposure on Thai vegetable farmers. Toxics. (2023) 11:707. doi: 10.3390/toxics11080707, PMID: 37624212 PMC10458049

[29] He J Han R Yu G Lavin MF Jia Q Cui P. Epimedium polysaccharide ameliorates benzene-induced aplastic anemia in mice. Evid Based Complement Alternat Med. (2020) 2020:1–12. doi: 10.1155/2020/5637507, PMID: 32256652 PMC7106868

[30] Ngatuni D Wairagu P Jillani N Isaac AO Nyariki JN. A glyphosate-based herbicide disrupted hematopoiesis and induced organ toxicities, ameliorated by vitamin B12 in a mouse model. Saudi J Biol Sci. (2022) 29:103278. doi: 10.1016/j.sjbs.2022.03.028, PMID: 35401022 PMC8987997

[31] Raiten DJ Moorthy D Hackl LS Dary O. Exploring the anemia ecology: a new approach to an old problem. J Nutr. (2023) 153:S1–6. doi: 10.1016/j.tjnut.2023.07.016, PMID: 37778890 PMC10797548

[32] Abd Rahman R Idris IB Md Isa Z Abd RR. The effectiveness of a theory-based intervention program for pregnant women with anemia: a randomized control trial. PLoS One. (2022) 17:e0278192. doi: 10.1371/journal.pone.0278192, PMID: 36473006 PMC9725169

[33] Reist C Petiwala I Latimer J Raffaelli SB Chiang M Eisenberg D. Collaborative mental health care: a narrative review. Medicine. (2022) 101:e32554. doi: 10.1097/md.0000000000032554, PMID: 36595989 PMC9803502

[34] Cao B Wang D Wang Y Hall BJ. Patient expectation in China: exploring patient satisfaction in online and offline patient-provider communication. Front Psychol. (2022) 13:888657. doi: 10.3389/fpsyg.2022.888657, PMID: 35756275 PMC9226754

[35] Tarım H Öz F. Thalassemia major and associated psychosocial problems: a narrative review. Iran J Public Health. (2022) 51:12–8. doi: 10.18502/ijph.v51i1.8287 35223621 PMC8837879

[36] Alasmari A Zhou L. Share to seek: the effects of disease complexity on health information-seeking behavior. J Med Internet Res. (2021) 23:e21642. doi: 10.2196/21642, PMID: 33759803 PMC8074994

[37] Wong MCS Wong ELY Huang J Cheung AWL Law K Chong MKC. Acceptance of the COVID-19 vaccine based on the health belief model: a population-based survey in Hong Kong. Vaccine. (2021) 39:1148–56. doi: 10.1016/j.vaccine.2020.12.083, PMID: 33461834 PMC7832076

[38] Schaeffer D Berens EM Vogt D Gille S Griese L Klinger J. Health literacy in Germany - findings of a representative follow-up survey. Dtsch Arztebl Int. (2021) 118:723–8. doi: 10.3238/arztebl.m2021.0310, PMID: 34551856 PMC8820084

[39] van Haalen H Jackson J Spinowitz B Milligan G Moon R. Impact of chronic kidney disease and anemia on health-related quality of life and work productivity: analysis of multinational real-world data. BMC Nephrol. (2020) 21:88. doi: 10.1186/s12882-020-01746-4, PMID: 32143582 PMC7060645

[40] Ram D Bheemaraju SP Alammar MA. Explanatory models and their relationship with drug attitude in patients with depression in South India. Indian J Psychol Med. (2023) 45:53–8. doi: 10.1177/02537176221098329, PMID: 36778620 PMC9896122

[41] Bergen N Labonté R. "everything is perfect, and we have no problems": detecting and limiting social desirability Bias in qualitative research. Qual Health Res. (2020) 30:783–92. doi: 10.1177/1049732319889354, PMID: 31830860

