# Supplementary material for: Knowledge, attitudes, and practices among patients with anemia towards disease management
Source: Front Public Health. 2024 May 21;12:1380710. doi: 10.3389/fpubh.2024.1380710 (PMC11149555; doi:10.3389/fpubh.2024.1380710)
Supplement: Supplementary file 1 [file Table_1.docx]

**Table S1. Knowledge of patients with anemia**

|  | **Correct N (%)** |
| --- | --- |
| K1. Anemia refers to a reduction in hemoglobin levels in the peripheral blood of the human body. (Correct) | 363(91.67) |
| K2. Imbalanced diet, poor cooking habits, and gastrointestinal diseases can lead to insufficient intake of iron, folic acid, and vitamin B12, causing anemia. (Correct) | 325(82.07) |
| K3. Chemical substances such as benzene, herbicides, and pesticides can induce anemia. (Correct) | 151(38.13) |
| K4. Viral infections may trigger anemia. (Correct) | 350(88.38) |
| K5. Chronic diseases and malignant tumors may induce anemia. (Correct) | 362(91.41) |
| K6. The skin of individuals with anemia generally appears pale. (Correct) | 374(94.44) |
| K7. Anemic individuals may experience symptoms such as headaches, tinnitus, and cerebral hypoxia. (Correct) | 365(92.17) |
| K8. Anemic individuals may exhibit increased respiration and heart rate, and prolonged anemia can lead to anemic heart disease. (Correct) | 327(82.58) |
| K9. Anemic individuals may suffer from loss of appetite, nausea, abdominal bloating, and other symptoms. (Correct) | 340(85.86) |
| K10. Anemia in women of childbearing age may lead to abnormal menstrual flow, and severe anemia can result in reduced sexual function. (Correct) | 340(85.86) |
| ~~K11.~~ ~~Individuals with anemia should seek immediate medical attention from a hematologist. (Trap question)~~ (Correct) | ~~322(81.31)~~ |
| K12. Although the causes of anemia may vary, the treatment principles are the same. (Incorrect) | 175(44.19) |
| ~~K13.~~ ~~Individuals with anemia shouldn’t seek immediate medical attention from a hematologist. (Trap question)~~ (Incorrect) | ~~322(81.31)~~ |
| K14. Rest and correction of the anemic state are generally the initial measures in the treatment of anemia. (Correct) | 352(88.89) |
| K15. Treatment options for anemia may include iron supplements, blood transfusions, and hematopoietic growth factors. (Correct) | 368(92.93) |
| K16. A high-fat diet or the consumption of certain vegetables and fruits (such as peach kernels, apricot kernels, etc.), strong tea, or coffee may inhibit iron absorption. Therefore, individuals with anemia should pay attention to their diet in daily life. (Correct) | 351(88.64) |

**Table S2**. **Attitudes** **of patients with anemia**

|  | **Strongly agree N (%)** | **Agree N (%)** | **Neutral N (%)** | **Disagree N (%)** | **Strongly disagree N (%)** |
| --- | --- | --- | --- | --- | --- |
| A1. Are you willing to learn about self-management of anemia through various forms such as books, short videos, and communication with doctors? (Positive) | 121(30.56) | 185(46.72) | 47(11.87) | 33(8.33) | 10(2.53) |
| A2. Do you recognize the role of regular exercise, healthy diet, and a consistent daily routine in the prevention and management of anemia? (Positive) | 144(36.36) | 201(50.76) | 43(10.86) | 7(1.77) | 1(0.25) |
| A3. Do you believe that active self-management of the disease can improve the condition of anemia or reduce the likelihood of its occurrence? (Positive) | 126(31.82) | 224(56.57) | 39(9.85) | 7(1.77) | 0 |
| A4. Do you consider it highly important to follow medical advice, attend regular follow-up appointments, and take medication as prescribed? (Positive) | 213(53.79) | 169(42.68) | 13(3.28) | 1(0.25) | 0 |
| A5. Do you think it is crucial to closely monitor symptoms of anemia? (Positive) | 172(43.43) | 190(47.98) | 33(8.33) | 1(0.25) | 0 |
| A6. Does the physical and mental discomfort caused by anemia and the potential for serious complications make you anxious? (Negative) | 112(28.28) | 176(44.44) | 69(17.42) | 28(7.07) | 11(2.78) |
| A7. Do you have full confidence in your ability to strictly adhere to self-management practices for anemia? (Positive) | 106(26.77) | 197(49.75) | 86(21.72) | 6(1.52) | 1(0.25) |

**Table S3. Practices of patients with anemia**

|  | **Very compliant N (%)** | **Moderately compliant N (%)** | **Somewhat compliant N (%)** | **Not very compliant N (%)** | **Not at all compliant N (%)** |
| --- | --- | --- | --- | --- | --- |
| P1. In the past year, have you sought information about anemia? | 100(25.25) | 138(34.85) | 86(21.72) | 55(13.89) | 17(4.29) |
| P2. To better manage the disease, have you developed specific recipes or taken supplements recommended by your doctor to improve the condition of anemia? | 123(31.06) | 139(35.10) | 95(23.99) | 32(8.08) | 7(1.77) |
| P3. For better disease management, do you engage in regular physical activities or exercises according to the advice of doctors or healthcare professionals, as long as your physical condition allows? | 108(27.27) | 148(37.37) | 101(25.51) | 30(7.58) | 9(2.27) |
| P4. For better disease management, are you able to follow the medication treatment plan prescribed by your doctor or healthcare professional, including specified medication doses and timings? | 175(44.19) | 179(45.20) | 41(10.35) | 0 | 1(0.25) |
| P5. For better disease management, do you regularly schedule follow-up appointments at the hospital? | 208(44.19) | 150(37.88) | 36(9.09) | 2(0.51) | 0 |
| P6. In your daily life, do you remain vigilant about symptoms related to anemia? | 156(36.87) | 163(41.16) | 67(16.92) | 8(2.02) | 2(0.51) |
| P7. Do you pay attention to restricting physical activities in your daily life to prevent or alleviate anemia? | 146(36.87) | 183(46.21) | 57(14.39) | 8(2.02) | 2(0.51) |
| P8. When you realize that the disease is causing negative emotions such as depression and anxiety, do you actively seek psychological counseling or other forms of support? | 101(25.51) | 132(33.33) | 91(22.98) | 54(13.64) | 18(4.55) |
| P9. For better disease management, do you actively cooperate with the treatment of the underlying disease? | 201(50.76) | 167(42.17) | 27(6.82) | 1(0.25) | 0 |
| P10. For better disease management, do you engage in active communication with fellow patients with anemia, learning about effective treatment methods or lifestyles? | 151(38.13) | 148(37.37) | 77(19.44) | 13(3.28) | 7(1.77) |

**Table S4. Pearson’s analysis** **of KAP scores**

|  | **Knowledge** | **Attitudes** | **Practices** |
| --- | --- | --- | --- |
| **Knowledge** | 1 |  |  |
| **Attitudes** | 0.283 (P<0.001) | 1 |  |
| **Practices** | 0.236 (P<0.001) | 0.604 (P<0.001) | 1 |

**Table S5. Test results of the path analysis**

| Model paths | Direct Effect | | Indirect effect | |
| --- | --- | --- | --- | --- |
|  | β (95% CI) | P | β (95% CI) | P |
| A←K | 0.484 (0.363, 0.647) | 0.008 | - | - |
| P←K | 0.247 (-0.024, 0.520) | 0.131 | 0.579(0.434, 0.805) | 0.004 |
| P←A | 1.195 (1.062, 1.332) | 0.007 | - | - |
